# Identification of Seven-Gene Hypoxia Signature for Predicting Overall Survival of Hepatocellular Carcinoma

**DOI:** 10.3389/fgene.2021.637418

**Published:** 2021-04-09

**Authors:** Yuping Bai, Wenbo Qi, Le Liu, Jing Zhang, Lan Pang, Tiejun Gan, Pengfei Wang, Chen Wang, Hao Chen

**Affiliations:** ^1^Department of MR, Lanzhou University Second Hospital, Lanzhou, China; ^2^Department of Surgical Oncology, Lanzhou University Second Hospital, Lanzhou, China; ^3^The Key Laboratory of the Digestive System Tumors of Gansu Province, Second Hospital of Lanzhou University, Lanzhou, China

**Keywords:** HCC, hypoxia, gene signature, prognosis, bioinformatics

## Abstract

**Background:**

Hepatocellular carcinoma (HCC) is ranked fifth among the most common cancer worldwide. Hypoxia can induce tumor growth, but the relationship with HCC prognosis remains unclear. Our study aims to construct a hypoxia-related multigene model to predict the prognosis of HCC.

**Methods:**

RNA-seq expression data and related clinical information were download from TCGA database and ICGC database, respectively. Univariate/multivariate Cox regression analysis was used to construct prognostic models. KM curve analysis, and ROC curve were used to evaluate the prognostic models, which were further verified in the clinical traits and ICGC database. GSEA analyzed pathway enrichment in high-risk groups. Nomogram was constructed to predict the personalized treatment of patients. Finally, real-time fluorescence quantitative PCR (RT-qPCR) was used to detect the expressions of KDELR3 and SCARB1 in normal hepatocytes and 4 HCC cells. The expressions of SCARB1 in hepatocellular carcinoma tissue in 46 patients were detected by immunohistochemistry, and the correlation between its expressions and disease free survival of patient was calculated.

**Results:**

Through a series of analyses, seven prognostic markers related to HCC survival were constructed. HCC patients were divided into the high and low risk group, and the results of KM curve showed that there was a significant difference between the two groups. Stratified analysis, found that there were significant differences in risk values of different ages, genders, stages and grades, which could be used as independent predictors. In addition, we assessed the risk value in the clinical traits analysis and found that it could accelerate the progression of cancer, while the results of GSEA enrichment analysis showed that the high-risk group patients were mainly distributed in the cell cycle and other pathways. Then, Nomogram was constructed to predict the overall survival of patients. Finally, RT-qPCR showed that KDELR3 and SCARB1 were highly expressed in HepG2 and L02, respectively. Results of IHC staining showed that SCARB1 was highly expressed in cancer tissues compared to adjacent normal liver tissues and its expression was related to hepatocellular carcinoma differentiation status. The Kaplan-Meier survival showed a poor percent survival in the SCARB1 high group compared to that in the SCARB1 low group.

**Conclusion:**

This study provides a potential diagnostic indicator for HCC patients, and help clinicians to deepen the comprehension in HCC pathogenesis so as to make personalized medical decisions.

## Introduction

Hepatocellular carcinoma (HCC), characterized by high morbidity and mortality, poses a major challenge to global public health (El-Serag, 2011). At the time of diagnosis, most patients have lost the opportunity for curative treatment, including transplantation, resection or ablation. In addition, due to the high recurrence rate, patients receiving potential treatment still have a poor prognosis (Yang et al., 2019). If patients are at higher risk of recurrence, strict follow-up is required, and patients may also benefit from adjuvant therapy after cure, although no adjuvant therapy has hitherto been considered standard treatment (França et al., 2004; Wang et al., 2013). There has been no consensus on the exploitation to predict the prognosis of HCC, though a slew of attempts and efforts have been made. Previous studies have mostly used parametric prediction models constructed with clinical baseline characteristics (such as tumor size, cirrhosis, tumor number, and microvascular infiltration) and single-molecule biomarkers (such as alpha-fetoprotein [AFP] and Des-γ carboxyl-Carboxyl of enzyme) to predict the prognosis of HCC (AlSalloom, 2016). But recently, with the development of genome sequencing technology, the integration of prognostic gene signatures and traditional parameters in the prognosis of HCC has shown great advantages. Nevertheless, it is still of great necessity to make endeavor for the application of these neoteric genetic properties in clinical practice.

Hypoxia is one of the markers of tumor microenvironment. Due to insufficient blood supply, growing tumors often occur in a hypoxia state (Lou et al., 2017). Unlike healthy cells, tumors respond to low oxygen levels by initiating multiple adaptive behaviors (for example, angiogenesis, proliferation, and invasion) that ultimately promote a more aggressive tumor phenotype. For example, glioblastoma can cause extensive tissue hypoxia, which facilitates the induction and maintenance of malignant phenotypes. For the glioma group, tumor hypoxia is associated with anti-apoptosis, tumor recurrence, resistance to chemotherapy and radiotherapy, invasion potential and reduced patient survival (D’Alessio et al., 2019). In addition, previous studies have shown that nearly 50% of locally advanced breast cancers suffer from hypoxia, leading to failed chemoradiotherapy resistance (Vaupel et al., 2002). However, despite considerable efforts on the relationship between hypoxia and tumor, the prediction of the correlation between hypoxic-related gene expression and overall survival rate in HCC patients has not been reported.

In this study, seven hypoxia gene signatures associated with HCC prognosis were constructed using TGCA dataset and validated in the ICGC dataset. Through GSEA functional enrichment analysis, we examined the important role of the prognostic marker gene in the development and progression of HCC. The final results showed that the model had high reliability in predicting the prognosis of HCC patients, and could help clinicians better carry out individualized treatment. After these bioinformatics analyses, two genes not previously reported in HCC, KDELR3 and SCARB1, were selected to study their expression levels in normal hepatocytes L02, HCC cells SMMC-7721, HepG2, huh7, and SK-Hep-1, respectively.

## Materials and Methods

### Sample Collection

RNAseq data of this sample mainly comes from TCGA cancer database^1^ and ICGC international cancer database^2^, including TGCA dataset as a training set, and ICGC dataset as a verification set. In order to ensure the accuracy and reliability of the data results, the samples with incomplete clinical information and TCGA samples with survival time less than 30 days were eliminated, and finally retained 343 TCGA liver cancer samples and 231 ICGC samples. The 200 hypoxia-related genes were retrieved from Molecular Signatures Database (MSiDb v7.1) and named as HALLMARK_HYPOXIA.

### Prognostic Model Construction

In order to establish a reliable prognosis signature, we first use Univariate Cox regression analysis to screening the prognostic genes. We then using the random forest algorithm to make a feature selection using the “randomForestSRC” R package. We also applied the randomSurvivalForest algorithm to screening the importance of prognostic-related genes. The genes with a relative importance >0.4 as the final signature. We then using the multivariate cox regression analysis to established an prognostic model based on these genes, and the risk score for each patients was calculated according to therisk formula = ∑Coef_gene_×Exp_genes_, where Coef_*gene*_ represent the coefficient of each prognostic gene, the Exp_*genes*_ represents each gene expression (Dong and Mingjun, 2019). The patients were further divided into high-risk and low-risk groups based on the median risk score in the TCGA and ICGC dataset, respectively.

### Prognostic Model Evaluation

Kaplan-Meier (KM) curve was used to analyze and compare the survival differences between the high-risk and low-risk groups in the prognostic model. Then, ROC curve was used to evaluate the specificity and sensitivity of the prognostic model by using the “survivalROC” R package. In addition, the KM curve was also used to assess the association between prognostic model risk values and clinical traits. To further assess whether the prognostic model could be used as an independent predictor, univariate and multivariate Cox regression analyses were used to evaluate clinical traits and risk values in training sets and external validation sets, and it was found that the prognostic model could be used as an independent predictor. Furthermore, we constructed a nomogram and calibration curve related to these independent predictors for personalized independent survival prediction through the “rms” R package.

### GSEA Enrichment Analysis

In order to assess the metabolic pathways involved in the prognostic model, we used GSEA enrichment analysis to assess the enrichment pathways of patients in the high-risk and low-risk groups. We are going to use c2.cp.kegg.v7.1.symbols.gmt as background. The screening of significant pathways was considered to be statistically significant with *p*-value < 0.05 and error discovery rate FDR < 0.05.

### Cell Culture

Human normal cell line L02, HCC cell lines SMMC-7721, Hep G2, huh7 and SK-Hep-1 were purchased from Cell Resource Center, PMUC (Beijing China). All cells were maintained in Dulbecco’s modified Eagle’s medium (DMEM; Gibco, United States) supplemented with 10% fetal bovine serum (FBS; Gibco), 1% penicillin and streptomycin (Gibco). The cells were cultured in a 5% CO2-humidified atmosphere at 37°C.

### RNA Extraction, Reverse-Transcription RNA, and Quantitative Real-Time Polymerase Chain Reaction

The total RNA was extracted from cell lines by using the TRIzol reagent (Invitrogen), reverse transcription was performed by using the PrimeScript RT reagent Kit (Takara, Japan) and cDNA was synthesized according to the manufacturer’s instructions. The qPCR assay was performed by LightCycler480 system (Roche, Switzerland) and SYBR Green (Takara).

SCARB1: Primer name(F): GGAGATCCCATCCCCTTCTAT, Primer name(R): CTGAACTCCCTGTACACGTAG.

KDELR3: Primer name(F): GAGGCTGAGACCATAACTA CTC, Primer name(R):AGAAATTCTCAGTCTGGTACCG.

### Patients and Specimens

A total 46 formalin-fixed and paraffin-embedded HCC tissue samples from patients who underwent curative surgical resection at the Lanzhou University Second Hospital (LanZhou, China), from 2016 to 2020, were included. The study protocol was approved by the Institutional Ethics Committee of Lanzhou University Second Hospital (Lanzhou, China) and the patients provided written informed consent regarding the use of their tissues. The main clinicopathological features of these patients are listed (Table 1). All patients underwent radical surgery. Median follow-up was 32 months. Disease free survival (DFS) was defined as time interval from operation date to recurrence.

**TABLE 1:** Major demographic and clinicopathological characteristics of hepatocellular carcinoma cases (*n* = 50).

Patients characteristic	Frequency (*n*)	Percentage (%)
**Age (years)**		
<65 years	18	36%
≥65 years	32	64%
**Sex**		
Male	36	72%
Female	14	28%
**Tumor size (cm)**		
≤5	13	26%
>5	37	74%
**Tumor number**		
Single	37	74%
Multiple	13	26%
**AFP (ng/ml)**		
≤200	28	56%
>200	22	44%
**Liver cirrhosis**		
No	26	52%
Yes	24	48%
**Grade**		
G1–2	29	58%
G3–4	21	42%
**TNM stage**		
Early (Stage I and II)	23	46%
Advanced (Stage III and IV)	27	54%
DFS (Median and range)	590 (0-28.0)	

*Values are expressed as median (range) or n (%). TNM, tumor-nodes-metastasis; AFP, α-fetoprotein; DFS, disease-free survival.*

### Immunohistochemical (IHC) Staining Procedure

Tissue microarray samples were cut into 4 um serial sections and then were placed in an oven at 67°C for 30 min, and further dewaxing in xylene and alcohol. Then tissue samples were treated with TE buffer (pH 9.3, 1 mM EDTA, and 10 mM Tris) at 98°C for 30 min. Next, tissue samples were immersed in 3% H2O2 in order to eliminate the endogenous peroxidase activity. Then samples were incubated with primary antibodies (SCARB1, 1:100, ABclonal, United States) in phosphate-buffered saline+Tween-20 (PBST) containing 3 mg/ml goat globulin (Sigma, St. Louis, MO, United States) for 60 min at room temperature (RT). Then anti-mouse/rabbit antibody (Envision plus, Dako) were used to incubated with tissue samples for 30 min at RT. Lastly, chromogenic agent 3, 3′-diaminobenzidine (Dako) was used to stain tissue samples.

We were examined and scored the IHC results. According to the positive cells’ proportion and the staining intensity, scores were assigned: [score 0], no or less than 5% positive cells; [score 1], 6–20% positive cells; [score 2], 21–50% positive cells; [score 3], more than 50% positive cells. The staining scores of 2 or 3 were considered as high expression, the staining results were scored as high and low.

### Statistical Analysis

Statistical software SPSS 20.0 was used for data analysis. Wilcoxon rank test analysis was used to compare different groups. *P* < 0.05 was considered statistically significant. The survival difference was analyzed using the Kaplan-Meier curve analysis and log-rank test analysis.

## Results

### Establishment and Validation of Hypoxia-Related Prognostic Models

**FIGURE 1:**
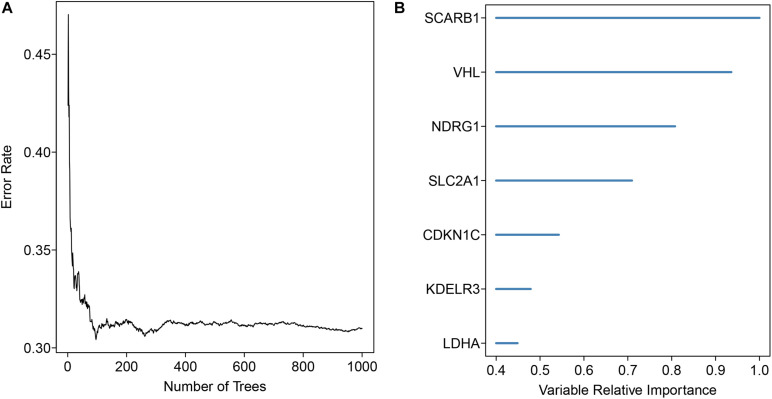
Gene signature selection using the forest analysis. **(A)** The distribution of the error rate **(B)** The importance of the signature genes was ranked through the random forest analysis in HCC.

**FIGURE 2:**
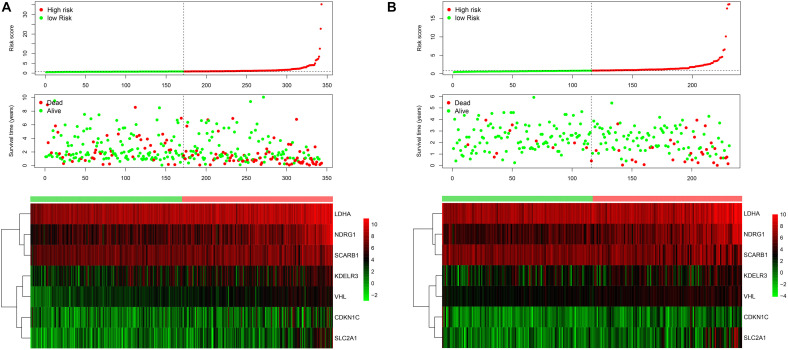
The risk score distribution for each patient was showed in the training dataset **(A)** and external validation dataset **(B)**. The upper panel represent the risk score distribution, the middle panel showed the cases distribution and the lower panel. Exhibited the gene expression.

**FIGURE 3:**
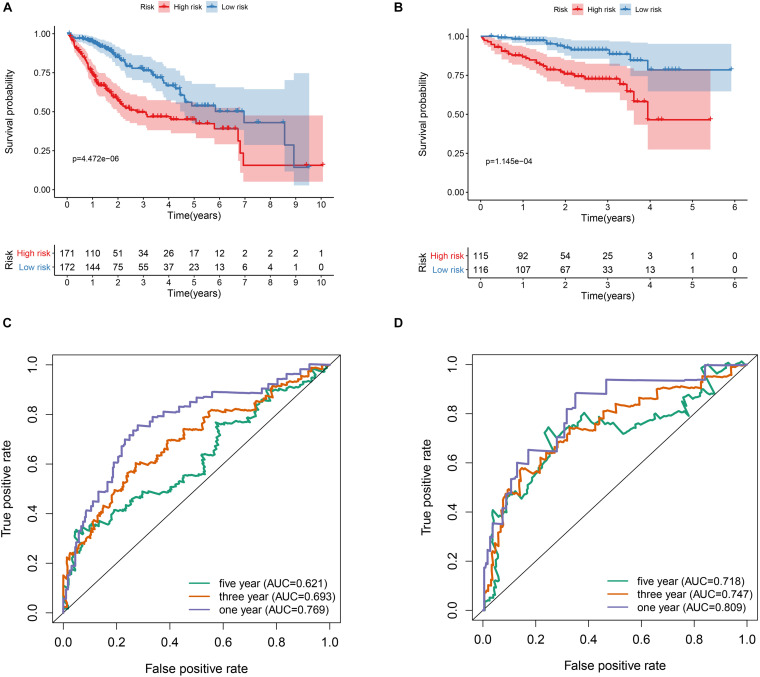
Prognostic value of the hypoxia risk signature in HCC. **(A)** Kaplan-Meier curve analysis of the hypoxia risk signature in the TCGA dataset. **(B)** Kaplan-Meier curve analysis of the hypoxia risk signature in the ICGC dataset. **(C)** ROC curve analysis of the hypoxia risk signature of the 1-, 3-, and 5-year in the TCGA dataset. **(D)** ROC curve analysis of the hypoxia risk signature of the 1-, 3-, and 5-year in the ICGC dataset.

343 patients with liver cancer and 200 genes associated with hypoxia were used to identify the prognostic model. Using univariate cox regression analysis, 79 survival-related hypoxic genes were selected forest and then the random forest algorithm was used for feature selection. We identified the genes of the relative important gene >0.4 was identified as the final feature (Figures 1A,B). In view of these characteristics, we used multivariate Cox regression analysis to construct the prognosis model containing seven hypoxic gene-related genes. Through the correlation coefficient, the risk formula was constructed, as follows: *r* risk score = LDHA × 0.000812695+ KDELR3 × 0.000649537+ CDKN1C × 0.002057653+ SLC2A1 × 0.004190531+ NDRG1 × 0.001235623+ VHL × 0.023669962+ SCARB1 × 0.00060351. Through the risk formula, the risk values of each patient in the training set and external validation set were calculated, and the patients were further divided into high-risk group and low-risk group on the basis of the median risk value. We found the number of death toll from the high-risk group of patients is significantly higher than low-risk group (Figures 2A,B), moreover, the KM curve analysis that according to the results of the survival of high and low risk group has obvious differences, the survival rate of patients with low risk is far higher than the risk group (*P* < 0.05) (Figures 3A,B), in addition, the results show that the training sample set and validation set outside the ROC curve prognosis is of high accuracy (Figures 3C,D).

### Independent Assessment of Prognostic Model

**FIGURE 4:**
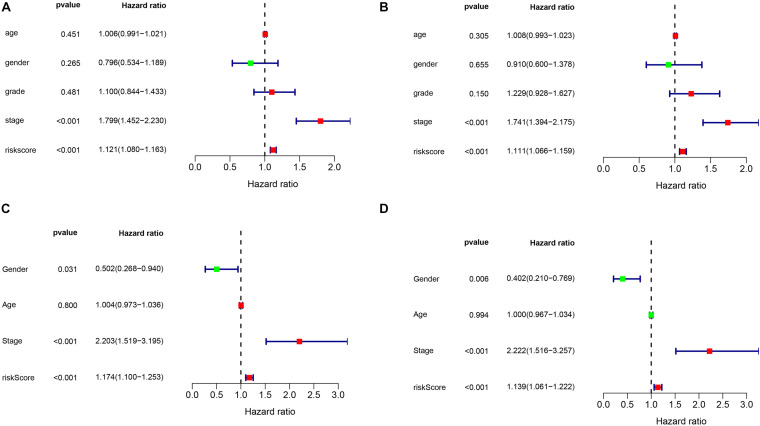
The independence identification of the risk model. Univariate cox regression analysis and multivariate cox regression analysis was performed in the TCGA dataset **(A,B)** and ICGC dataset **(C,D)**, respectively.

To assess the prognostic independence of this prognostic model in both the training set and the external validation set samples. We then performed the univariate cox regression analysis and multivariate cox regression analysis in both data sets. As showed in Figures 4A,B, we could discovered that the risk model can serve as an independent prognostic factor in HCC. In addition, we also observed that the risk model could act as an independent prognostic factor in the external validation dataset by performing univariate cox regression analysis (Figure 4C) and multivariate cox regression analysis (Figure 4D).

### Association Between Prognostic Models and Clinical Cause Groups

**FIGURE 5:**
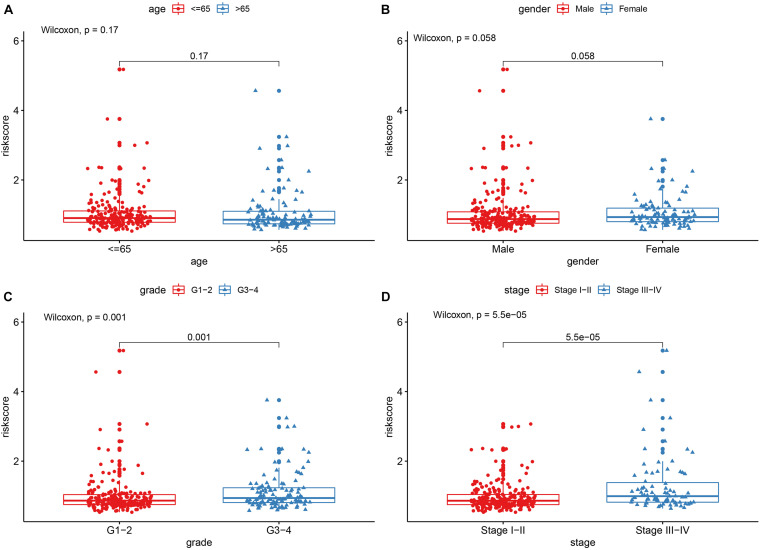
The correlations between the risk model and clinical factors. **(A)** Age. **(B)** Gender. **(C)** Grade. **(D)** Stage.

**FIGURE 6:**
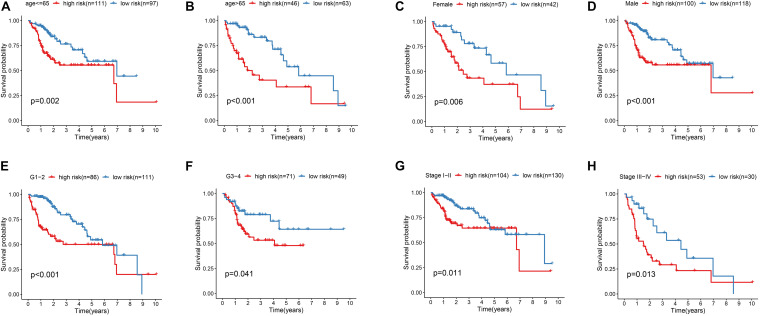
Kaplan-Meier survival curves for the high- and low-risk groups stratified by clinical factors. **(A,B)** Age. **(C,D)** Gender. **(E,F)** Grade. **(G,H)** Stage.

In order to explore the association between prognostic models and clinical traits, we first assessed the distribution of risk values in clinical traits. As showed in Figures 5A,B, the risk score distribution have no significantly difference in age and gender. While risk values for G3-4 were significantly higher than G1–2 (*P* < 0.05) (Figure 5C). In addition, the risk values for stage III–IV were significantly higher than that of Stage I-II (Figure 5D). These results suggest that a higher risk score is associated with a higher degree of HCC malignance. Therefore, this prognostic model can accurately predict the progression of HCC. In addition, in order to study the prognostic value of the model stratified by clinicopathological variables for HCC patients, stratified analysis was conducted for HCC patients according to age (Figures 6A,B), gender (Figures 6C,D), grade (Figures 6E,F), and stage. For all the different stratifications, the Overall Survival (OS) time was significantly shorter in the high-risk group than in the low-risk group (Figure 6). These results suggest that the prognostic model can predict the prognosis of HCC patients without considering clinicopathological variables.

### The Construction and Verification of Nomogram in TCGA Data Set and ICGC Data Set

**FIGURE 7:**
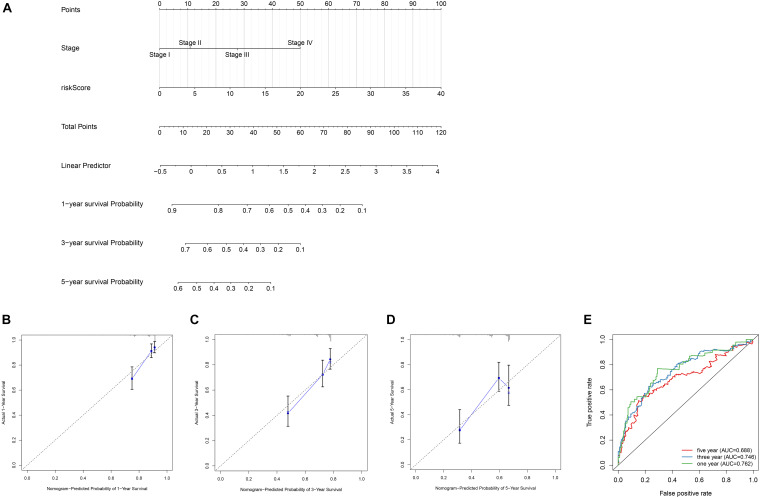
Construction of the nomogram and calibration curve plot. **(A)** Nomogram construction based on the independent clinical factors including stage and risk score. **(B)** Calibration curve plot for predicting 1-year OS in TCGA dataset. **(C)** calibration curve plot for predicting 3-year OS in TCGA dataset. **(D)** Calibration curve plot for predicting 5-year OS in TCGA dataset. **(E)** ROC curves analysis of the nomogram in 1-, 3-, and 5-year OS.

In order to establish quantitative prognostic methods for HCC, nomogram was established by independent prognostic factors and prognostic models of two data sets. Based on multivariate Cox analysis, point ratios in nomogram were used to assign points. We drew a horizontal line to determine the points for each variable, calculated the total points for each patient by adding the points for all variables, and normalized it to a distribution of 0 to 100. By drawing a vertical line between the total point axis and each pre-posterior axis, we can calculate the estimated 1-, 3-, and 5-year survival rates of HCC patients, which may be helpful for practitioners to conduct clinical decisions about the prognosis of HCC patients. In addition, we evaluated the accuracy and consistency of the nomogram by performing the ROC curve and the calibration curve, respectively (Figure 7).

### Functional Enrichment Analysis of Prognostic Model

**FIGURE 8:**
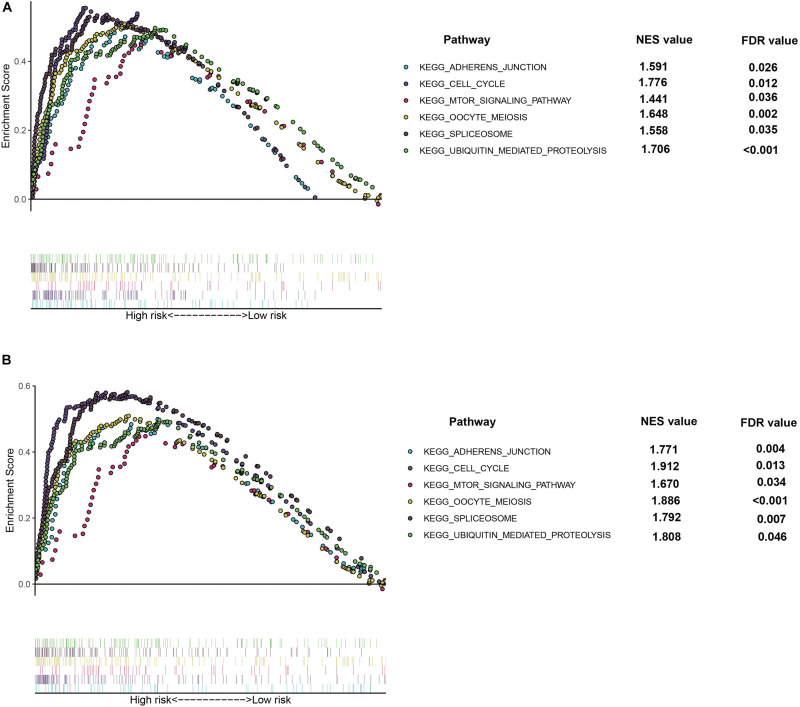
Gene Set Enrichment Analysis (GSEA) of the hypoxia risk signature in the high risk group of TCGA **(A)** and ICGC **(B)** dataset, respectively.

In order to further explore the potential function and role of prognostic models in HCC, GSEA enrichment analysis was used for enrichment analysis of high and low risk groups. The results showed the cell cycle, MTOR signaling pathways, OOCYTEMEIOSIS and UBIQUITINMEDIATESPROTEOLYSIS pathway were significantly enriched in the high-risk group (Figure 8).

### Expression Levels of kdelr3 and SCARB1

**FIGURE 9:**
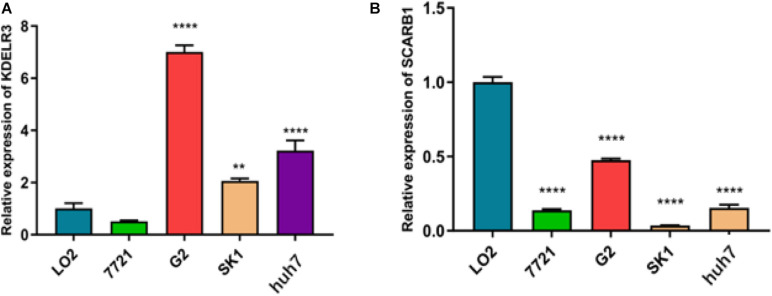
Relative expression of KDELR3 **(A)** and SCARB1 **(B)** 7721 represent SMMC-7721,G2 represent Hep G2,SK1 represent SK-Hep-1 (**p < 0.0016,****p < 0.0001).

To better explain the biological function of these genes in the pathogenesis and development of HCC, we selected two genes not reported in HCC studies for RT-qPCR to study the differences in their expression levels. We found that the expression of KDELR3 was the highest in G2, while the lowest in 7721, L02 was the highest in SCARB1, and SK-Hep-1 was the lowest (*p* < 0.05; Figure 9).

### Survival Analysis

**FIGURE 10:**
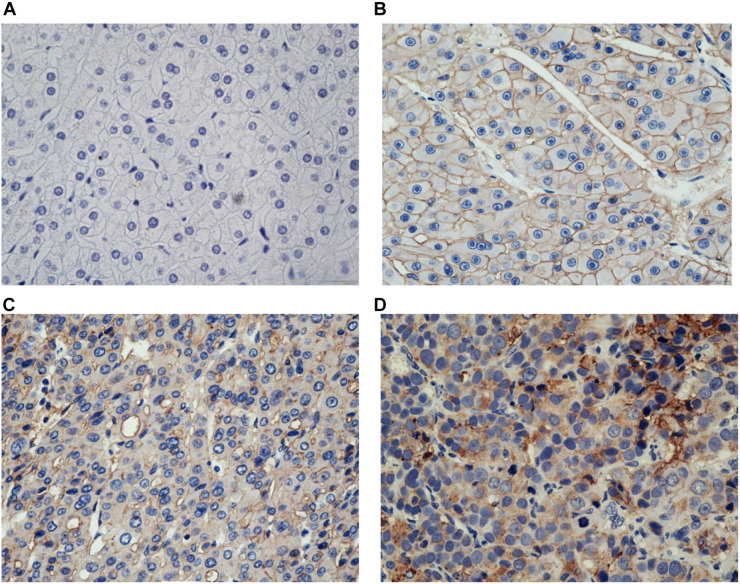
Immunostaining of SCARB1 in HCC or the control tissue. **(A)** Negative staining of SCARB1 in normal tissue adjacent to HCC (400 magnification); **(B)** Positive staining of SCARB1 in well differentiated HCC tissue (400 magnification); **(C)** Expression of SCARB1 in moderate differentiated HCC tissue (400 magnification); and **(D)** Expression of SCARB1 in poor differentiated HCC tissue (400 magnification). SCARB1 = scavenger receptor class B member 1.

**FIGURE 11:**
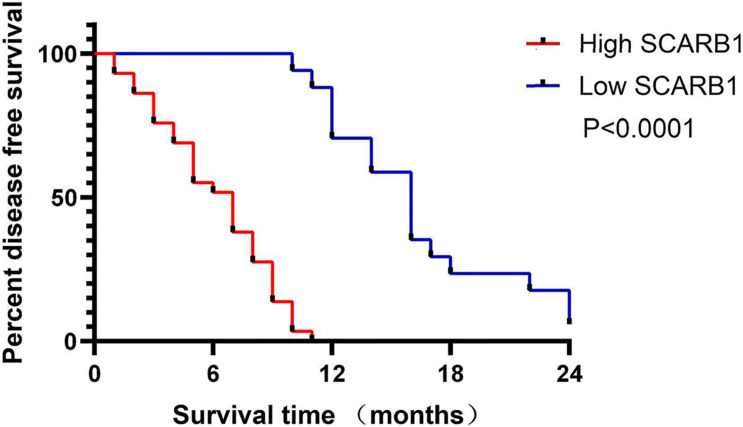
Kaplan-Meier analysis of the survival rate of patients with HCC. Disease-free survival of all patients in relation to SCARB1 expression (*p* < 0.0001).

For the 46 HCC cases, the median DFS was 9.0 months (range, 28 days-28.0 months). As expected, several clinical factors were associated with the DFS of HCC patients, including tumor Grade, TNM stage (p < 0.001 for all)(Table 2). Furthermore, SCARB1 was highly expressed in cancer tissues and the positive expression rate increased with the degrade of differentiation (Figure 10). The Kaplan-Meier survival was analyzed to study the effect of SCARB1 on HCC patient survival; it revealed that a poor percent survival in the SCARB1 high group compared to that in the SCARB1 low group (P < 0.001) (Figure 11).

**TABLE 2:** The correlation between SCARB1 and clinicopathological characteristics in HCC.

	SCARB1		
Patients characteristic	Low (*n* = 17)	High (*n* = 29)	*P*-value
Age (years)			0.232
<65	5	9	
≥65	12	20	
Sex			0.232
Male	12	20	
Female	5	9	
Tumor size (cm)			0.265
≤5	9	14	
>5	8	15	
Tumor number			0.568
Single	13	24	
Multiple	4	5	
AFP (ng/ml)			0.075
≤200	11	14	
>200	6	15	
Liver cirrhosis			0.156
No	8	15	
Yes	9	14	
Grade			<0.001
G1–2	10	2	
G3–4	7	27	
TNM stage			<0.001
Early (Stage I and II)	13	6	
Advanced (Stage III and IV)	4	23	

*Values are expressed as n (%). TNM, tumor-nodes-metastasis; AFP, alpha-fetoprotein.*

### Univariate and Multivariate Analysis

In Univariate and Multivariate analysis, traditional clinicopathological features (including tumor size, Grade and TNM stage), SCARB1 were associated with DFS. The results show that Grade, TNM stage and SCARB1 are correlated with DFS respectively (P = 0.001, P = 0.036, P < 0.001) (Table 3).

**TABLE 3:** Multivariate analyses of factors associated with DFS.

	Univariate analysis			Multivariate analysis		
Clinicopatho logical factor	*P*-value	HR	95% CI	*P*-value	HR	95% CI
Age (<605 vs. ≥65 years)	0.348	1.004	0.981-1.020			
Sex (female vs. male)	0.281	0.768	0.540–1.198			
Tumor size (≤5 vs. >5)	0.679	0.789	0.343-2.160	0.281	1.452	0.712-2.654
Tumor number (single vs. multiple)	0.310	0.378	0.131-1.418			
AFP (≤200 vs. >200)	0.097	1.812	0.891-3.189			
Liver cirrhosis (no vs. yes)	0.058	0.813	0.514–0.868			
Grade(G1–2 vs. G3–4)	0.041	1.177	0.850-1.530	0.036	1.102	0.618-1.890
TNM stage (early vs. advanced)^*a*^	0.038	1.755	1.465–2.437	<0.001	1.812	0.944–3.828
SCARB1 expression (low vs. high)	0.005	1.002	1.001-1.006	0.025	1.870	1.082–3.233

*^a^Early, TNM stage I/II; advanced, TNM stage III/IV. DFS, disease-free survival; SCARB1, scavenger receptor class B member 1; TNM, tumor-nodes-metastasis; HR, hazard ratio; CI, confdence interval.*

## Discussion

Hepatocellular carcinoma is one of the major health threats around the world, particularly in East Asia. Even after radical resection, the long-term outcome of HCC patients remains depressed (Mazzanti et al., 2016). Therefore, it is very important to develop a prognostic model suitable for HCC patients. Recently, with the improvement of genome sequencing, biochip and high-throughput sequencing technologies, more and more studies have applied bioinformatics methods to chip dataset analysis, which provides an effective new method for the diagnosis, treatment and prognosis of HCC. In this study, a total of 574 HCC samples were downloaded from TCGA and ICGC databases, using TCGA as the training set and ICGC as the test set. Through bioinformatics analysis, a prognostic model of HCC with seven genes associated with hypoxia was constructed for the first time. Our prognostic model can effectively stratify the survival of patients. We found the efficacy of our prognostic model in both the training set and the external validation set, suggesting that the model has strong prognostic value. In addition, the prognostic model showed a significant correlation with clinicopathological factors, further supporting the robustness of the prognostic role of our model. In addition, univariate and multivariate Cox regression analysis were used to validate our prognostic model as an independent predictor. Nomogram of independent predictors (staging and prognostic models) were constructed and showed that the model performed well in predicting 1-, 3-, and 5-year OS, which may be useful for planning short-term follow-up for individual treatment. In summary, the predictive prognostic value of our signatures is greatly reflected in these results, but it is worth noting that only two databases were selected. To validate the model on a large scale, the signatures need to be validated in a more independent queue.

Among the seven genes in the prognostic model that we constructed, LDHA (lactate dehydrogenase A) is A crucial REDOX enzyme in the glycolysis pathway in organisms, which can reversely catalyze the oxidation of lactic acid to pyruvate, and this catalytic reaction is the final product of anaerobic glycolysis (Guo et al., 2019). In addition, LDHA can be used as a possible prognostic marker for lung adenocarcinoma survival, and the high expression of LDHA is associated with poor prognosis (Yu et al., 2020). NDRG1 is a known metastasis inhibitor in a variety of cancers, participating in embryogenesis, cell growth, lipid biogenesis, stress response, and immunity. During metastasis, tumor growth and invasion require angiogenesis, and overexpression of NDRG1 is associated with a decrease in pro-angiogenic factors, resulting in a decrease of angiogenesis in pancreatic cancer (Hosoi et al., 2009). The expression levels of NDRG2 and LDHA are closely related to the prognosis of HCC patients and can be used as prognostic markers (Guo et al., 2019). KDELR3 is the third confirmed member in the KDEL familys, which encodes proteins associated with the endoplasmic reticulum (ER). Reports have shown previously that KDELR3 expression in arteriosclerosis macrophages could be obviously differ from that in non-arteriosclerosis tissues, and the higher expression level in non-arteriosclerosis tissues, which can be used as a potential prognostic factor (Huang et al., 2019). CDKN1C, DKN1C, also known as p57Kip2, are cyclin-dependent kinase inhibitors. The gene encoding CDKN1C is located on chromosome 11p15.5.CDKN1C belongs to the kinase inhibitor protein/CDK interacting protein (Kip/Cip) family, which consists of three members, namely CDKN1A/p21Cip1, CDKN1B/p27Kip1, and CDKN1C/p57Kip2. Previously, CDKN1C was identified as a tumor suppressor gene with decreased expression in various cancers including HCC, colorectal cancer and ovarian cancer (Peng et al., 2015). Thus, upregulation of CDKN1C leads to inhibition of markers involved in cell growth, differentiation, cell death, and angiogenesis in malignant tumors. SLC2A1 has been extensively studied as a major glucose transporter and has been identified as a possible prognostic factor for several cancers, including HCC and NSCLC et al. (Shang et al., 2020). The absence of von Hippel-Lindau (VHL), a tumor suppressor gene, is a hallmark of clear cell renal carcinoma. In addition, VHL inactivation leads to constitutive activation of hypoxia-inducing factor (HIF) HIF-1 and HIF-2 and their downstream targets (including pro-angiogenic factors VEGF and PDGF), while the activation of HIF and its downstream targets induces tumor formation (Gossage et al., 2015). SCARB1 (Scavenger receptor class B member 1) is a protein-coding gene. The related pathways include lipoprotein metabolism and folic acid metabolism. Studies have also found that SCARB1 can be a potential target for prostate cancer (Gordon et al., 2019).

The relationship between cell cycle and malignancy is now fully established, and our GSEA results show that the associated pathways are enriched in high-risk patients. The activation of mTOR related pathways can promote the vitality and motility of HCC cell lines (Wu et al., 2018). Enrichment of The OOCYTEMEIOSIS pathway was found in bladder cancer and cervical cancer (Gao et al., 2018; Wu et al., 2019). UBIQUITINMEDIATESPROTEOLYSIS related pathways plays an important role in the disease progression of colorectal cancer and non-small cell lung cancer (Lu et al., 2018; Wu et al., 2020). These three pathways were enriched in our high-risk patient group.

Although we have made a lot of efforts to study the prognostic model, there are still many defects. The clinical information of external validation set and test set is not fully matched, leading to the omission of partial clinical information and the inability to fully understand the correlation between the prognostic model and clinical practice. Moreover, none of genes have been verified, because some of them have been studied in HCC. In view of this, two genes, KDELR3 and SCARB1, have not been studied in HCC, laying a foundation for further studies in the future. In the present study, we have verified the RNA expression level of KDELR3 and SCARB1, protein expression level of SCARB1 and its percent survival, which verified our prognostic model. And more research is needed.

In conclusion, a prognostic model consists of seven genes associated with hypoxia was constructed to provide potential biomarkers for the prognosis of HCC, which contributed to the understanding of the underlying HCC pathogenesis.

## Data Availability Statement

The original contributions presented in the study are included in the article/supplementary material, further inquiries can be directed to the corresponding author/s.

## Author Contributions

HC designed this study. YB and WQ wrote the manuscript. LL and JZ collected the data. LP and TG analyzed the data. PW revised the manuscript. All authors approved the final version for submission.

## Conflict of Interest

The authors declare that the research was conducted in the absence of any commercial or financial relationships that could be construed as a potential conflict of interest.

## Footnotes

1 https://portal.gdc.cancer.gov/

2 https://ICGC.org/

## Abbreviations

TCGA: the cancer genome atlas
ICGC: international cancer genome consortium
KM: Kaplan–Meier
qRT-PCR: quantitative reverse transcription polymerase chain reaction
HCC: hepatocellular carcinoma
GSEA: gene set enrichment analysis
ROC: receiver operating characteristic
